# HIV Testing and Preventive Services Accessibility Among Men Who Have Sex With Men at High Risk of HIV Infection in Beijing, China

**DOI:** 10.1097/MD.0000000000000534

**Published:** 2015-02-13

**Authors:** Yuejuan Zhao, Li Zhang, Heng Zhang, Dongyan Xia, Stephen W. Pan, Hai Yue, Hongyan Lu, Hui Xing, Xiong He, Yiming Shao, Yuhua Ruan

**Affiliations:** From the Beijing Center for Disease Control and Prevention (YZ, LZ, DX, HY, HL, XH); State Key Laboratory for Infectious Disease Prevention and Control, National Center for AIDS/STD Control and Prevention, Chinese Center for Disease Control and Prevention, Collaborative Innovation Center for Diagnosis and Treatment of Infectious Diseases, Beijing, China (HZ, HX, YS, YR); The School of Population and Public Health University of British Columbia, Vancouver, BC, Canada (SWP).

## Abstract

The HIV epidemic among men who have sex with men (MSM) has been increasing at an alarming rate in most areas of China in recent years. Many Chinese MSM still lack sufficient access to HIV prevention services, despite ongoing scale-up of comprehensive HIV testing and intervention services. The purpose of this study was to investigate utilization of HIV testing and prevention services, and related factors that influence the MSM people to access HIV test or other services to prevent HIV among MSM in Beijing, China.

Three successive cross-sectional surveys of MSM were conducted in Beijing from September 2009 to January 2010, September 2010 to January 2011, and September 2011 to January 2012. Demographic and behavioral data were collected and analyzed. Blood samples were tested for HIV and syphilis. Three models were established to analyze factors associated with HIV testing and preventive services.

Of the 1312 participants, prevalence of HIV and syphilis was 7.9% and 15.4%, respectively. Sixty-nine percent ever had an HIV test, 56.2%, 78.7%, and 46.1% received HIV test, free condom/lubricants, and sexually transmitted infection services in the past 12 months (P12M), respectively. MSM with larger social networks and who knew someone infected with HIV were more likely to receive HIV testing and preventive services; lower degrees of stigma and discriminatory attitudes toward HIV/AIDS were positively associated with having an HIV test, whereas unprotected anal intercourse in the past 6 months (P6M) was associated with less preventive services participation. The most reported barriers to HIV testing were fear of testing HIV positive (79.3%) and perceiving no risk for HIV (75.4%). Almost all participants felt that ensuring confidentiality would encourage more MSM to have an HIV test. The two main reasons for not seeking HIV test was not knowing where to go for a test (63.2%) and perceiving low risk of HIV infection (55.1%).

Given a high prevalence of HIV, syphilis, and risky behaviors and a relatively low HIV testing rate among MSM in Beijing, more efforts are urgently needed to address barriers to HIV testing and improve accessibility of prevention services.

## INTRODUCTION

According to 2011 estimates, there are 780 000 people currently living with HIV/AIDS in China, accounting for 0.057% of the Chinese population.^1,2^ Although the HIV epidemic in China was initiated by sharing needles among intravenous drug users (IDUs) and spread among plasma donors in certain geographic regions thereafter, sexual transmission, especially homosexual transmission among men who have sex with men (MSM), has been increasing at an alarming rate in most areas of China in recent years.^3,4^ Sentinel surveillance shows that the overall HIV prevalence among MSM increased from 0.9% in 2003 to 6.3% in 2011.^5^

To cope with the ever-growing epidemic among MSM, both the Chinese government and community have taken steps to provide comprehensive HIV/AIDS prevention and intervention services to this hidden population. Such measures include peer education, provision of free condoms and lubricants, free voluntary HIV counseling and testing (VCT), and sexually transmitted infection (STI) diagnosis and treatment.^2,3,6^ These prevention measures have been proven to be effective in reducing risky behaviors and HIV transmission among Chinese MSM,^6–8^ and the coverage of HIV testing and other intervention services has increased substantially among Chinese MSM in recent years.^2,9^ However, due to the dual stigma and discrimination of both HIV and homosexuality that Chinese MSM might face, as well as other structural and psychological barriers, there remains a large proportion of MSM who are unable to access HIV testing and prevention services.^10,11^ Approximately, 61.1% to 87.0% of MSM infected with HIV remain undiagnosed.^12,13^

Given that comprehensive HIV testing and prevention services in China are continually being scaled-up but remain inaccessible to many Chinese MSM, greater understanding of barriers to these prevention services is urgently needed. We conducted the current study to investigate utilization of HIV testing and prevention services, and related factors that influence the MSM people to access HIV test or other services to prevent HIV among MSM in Beijing, China.

## METHODS

### Participants and Procedures

Three successive cross-sectional studies of MSM were conducted by using incentivized snowball sampling in Beijing, China from September 2009 to January 2010, September 2010 to January 2011, and September 2011 to January 2012. Participant eligibility criteria were as follows: male, self-reported sex with another man in the last 12 months, at least 18 years old, currently living or working in Beijing, and provision of written informed consent. To ensure maximum variation of each survey sample, initial participants were selected from diverse networks with respect to geography, venue (eg, bar, bathhouse, park, and internet), demographic characteristics, and subgroup membership (eg, money boys, bisexual, gay-identified vs non-gay identified); selection of network “seeds” was based on articulacy and reputation as motivated opinion leaders. Eligible participants completed a computer-assisted questionnaire, counseled for HIV testing, and then had their blood drawn. Participants were compensated 30 Chinese yuan (CNY) (∼5 USD) for participation, and 10 CNY (∼1.8 USD) for each eligible peer they referred. The study was approved by institutional review boards at the Chinese Center for Disease Control and Prevention—National Center for AIDS/STD Control and Prevention, Vanderbilt University, and University of California, San Francisco.

### Measures

Indicators and questionnaire development of this study were primarily based on our former qualitative study,^14^ and other researches published.^15–19^ Participants reported sociodemographic characteristics (age, ethnicity, education, marital status, employment, monthly income, and Beijing residence status), sexual behaviors (sexual orientation, number of sex partners in the past 6 months [P6M], unprotected anal intercourse [UAI] with up to 3 of their most recent male sex partners in P6M), perceived risk of HIV exposure from male partner, utilization of prevention services (accessing free condom/lubricants, HIV testing, or free STI diagnosis and treatment in the past 12 months [P12M), stigmatizing and discriminatory attitudes toward people living with HIV/AIDS (PLWHA), barriers and facilitators for HIV testing, and reasons for not seeking HIV testing.

Individual stigma and discriminatory attitudes toward PLWHA were scored by asking participants whether they agreed or disagreed with 22 statements. The scale was adapted from 2 pilot surveys conducted in Thailand and Zimbabwe,^20^ and includes 3 sections: shame, blame, and social isolation (10 items); discrimination (8 items); and equity (4 items). We included the first 2 sections in all 3 surveys (equity section was excluded in 2011), and therefore only the first 2 sections (18 items) were analyzed in this study. Items were summed to create a total score (items indicating positive attitudes toward PLWHA were reverse-coded), with a range of 18–36, in which a higher score represents a lower level of HIV/AIDS-related stigma and discrimination. Scale reliability was supported by a Cronbach's alpha value of 0.85.

Participants were asked whether they agreed or disagreed with each statement about barriers (13 statements) and facilitators (12 statements) for HIV testing, and reasons for not seeking HIV testing (10 statements).

### Statistical analysis

Observations of those who participated in >1 survey were excluded and the first observation for repeated individuals was reserved for final analysis. Since our focus was on identifying correlates of service utilization rather than temporal trends of service utilization, data from all 3 surveys were pooled into a single analytic dataset.

Data were analyzed using SAS 9.2 (SAS Institute Inc, Cary, NC, USA). Descriptive statistics were conducted for sociodemographic characteristics, sexual behaviors, perceived risk of HIV from male partners, utilization of prevention services, stigmatizing and discriminatory attitudes toward PLWHA, barriers and facilitators for HIV testing, reasons for not seeking HIV testing, and HIV and syphilis prevalence rates. Odds ratios (OR), 95% confidence intervals (CIs), and *P* values were calculated for factors associated with HIV testing and prevention services (3 models) using bivariate logistic regression. Variables with statistical significance (*P* < 0.05) in the univariate analysis were entered into the stepwise multivariate logistic regression analyses, and adjusted odds ratios (AORs) and 95% CIs were calculated. Moreover, enrollment year was included in multivariate analysis to adjust for the potential impact of time.

## RESULTS

### Sociodemographics Characteristics

A total of 500, 385, and 427 subjects were retained from each of the 3 surveys after excluding repeated observations of individuals who participated in >1 round.

Of the 1312 participants, the median age was 28 years, 94.4% were of Han ethnicity, 42.2% had college- or higher-level education, about a quarter (25.2%) had ever been married, 88.5% had a full time or part time job, 39.5% earned ≥3000 CNY (∼500 USD) per month in the last year, most participants (82.6%) did not have official Beijing residency status, and more than a half (52.5%) had lived <5 years in Beijing.

### Sexual Behavior and HIV Risk Perception

Among the 670 participants who reported ever having sex with a female, 44.3% reported sexual debut with a female at age ≤20. Among all participants, 48.9% of all participants reported sexual debut with a male at age ≤20, 15.2% reported having sex with female in P6M, about a half (47.3%) had no <3 male partners in P6M, 44% reported UAI with at least 1 of their last 3 partners in P6M, and >70% perceived small or no risk of HIV infection from sex with men.

### HIV/Syphilis Prevalence and Prevention Services Coverage

Prevalence of HIV and syphilis was 7.9% and 15.4%, respectively. Sixty-nine percent of men had previously received an HIV test, and 56.2% had an HIV test in P12M. The proportion of those who received free condom/lubricants and STI services in P12M was 78.7% and 46.1%, respectively. More than four-fifths of participants had received an HIV test, free condom/lubricants, or STI services in P12M (Table 1 ).

**TABLE 1:**
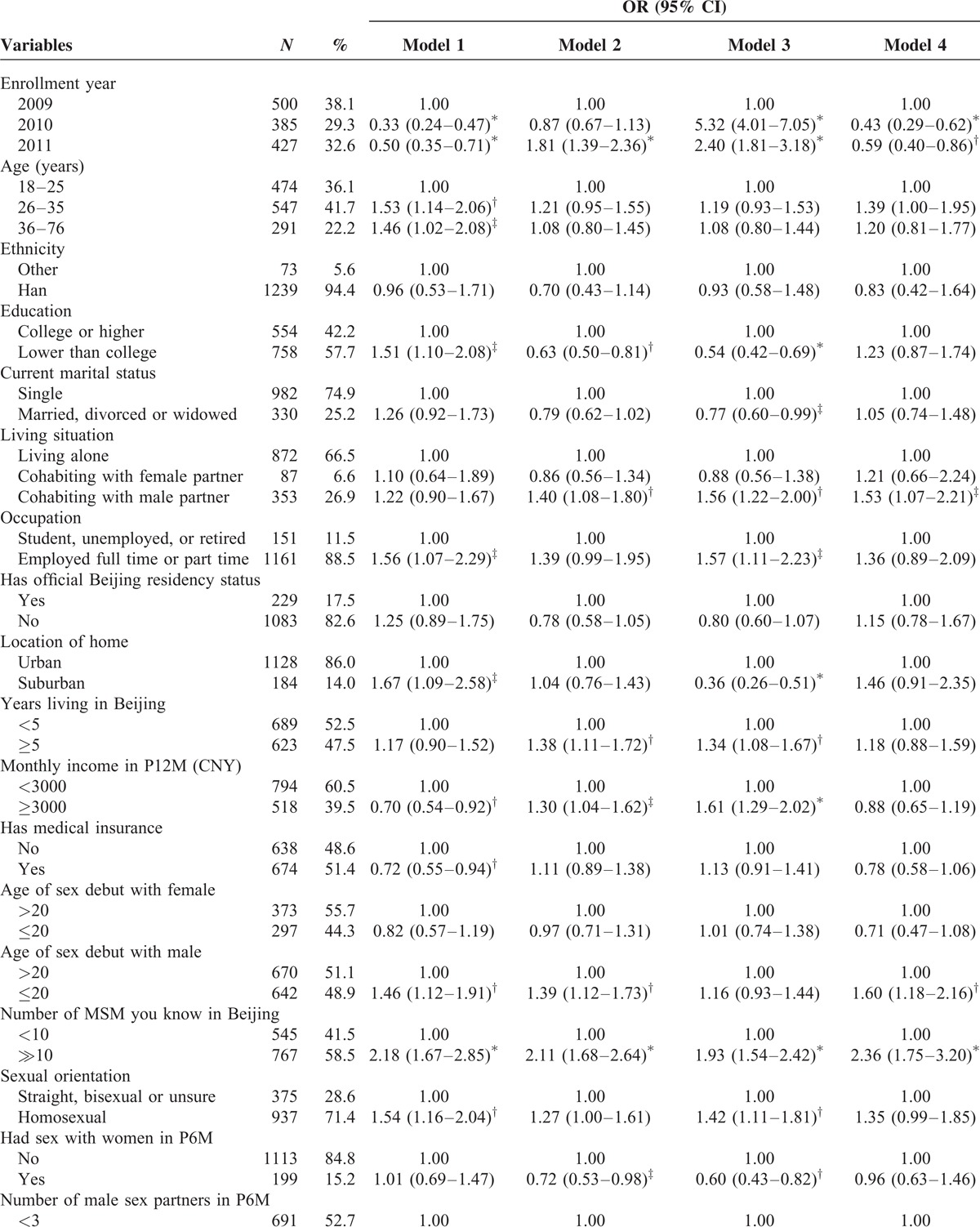
Characteristics of Participants and Bivariate Analysis of Association Between selected Variables and HIV/STI Testing and Intervention Services

**TABLE 1 (Continued):**
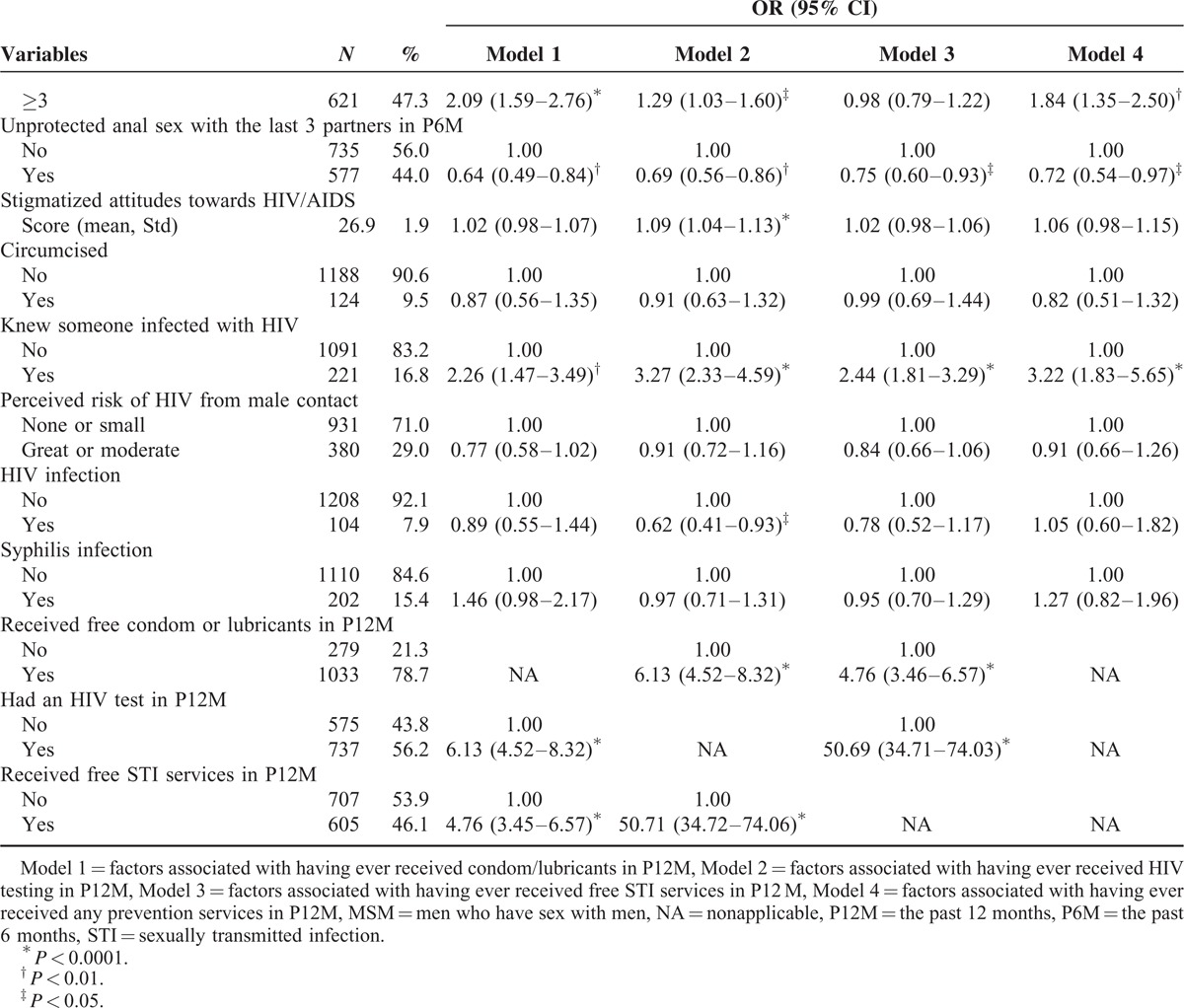
Characteristics of Participants and Bivariate Analysis of Association Between selected Variables and HIV/STI Testing and Intervention Services

### Predictors of HIV Testing and Prevention Services

Table 1  illustrated the results of bivariate analysis of the association between select variables and HIV/STI testing and intervention services.

Multivariate analysis indicated that participants enrolled in 2010 or 2011 (2010: AOR = 0.33, 95% CI: 0.23–0.47; 2011: AOR = 0.53, 95% CI: 0.36–0.77, vs 2009), earned ≥3000 CNY each month (AOR = 0.55, 95% CI: 0.40–0.76, vs <3000 CNY), or had UAI in P6M (AOR = 0.60, 95% CI: 0.45–0.79, v no UAI) were less likely to have obtained free condoms/lubricants in P12M. However, obtaining condoms/lubricants in the P12 M was positively associated with being ≥26 years (26–35: AOR = 1.77, 95% CI: 1.24–2.53; 36–76: AOR = 1.72, 95% CI: 1.14–2.60, vs 18–25 years), full or part-time employment (AOR = 2.02, 95% CI: 1.30–3.15, vs student, unemployed, or retired), having homosexual debut at age ≤20 years (AOR = 1.59, 95% CI: 1.15–2.20, vs >20 years), knowing ≥10 MSM in Beijing (AOR = 2.04, 95% CI: 1.52–2.75, vs <10), identifying as homosexual (AOR = 1.40, 95% CI: 1.03–1.91, vs straight, bisexual, or unsure), having ≥3 male partners in P6M (AOR = 1.63, 95% CI: 1.21–2.20, vs <3), and knowing someone infected with HIV (AOR = 1.97, 95% CI: 1.24–3.11) (Table 2, Model 1).

**TABLE 2:**
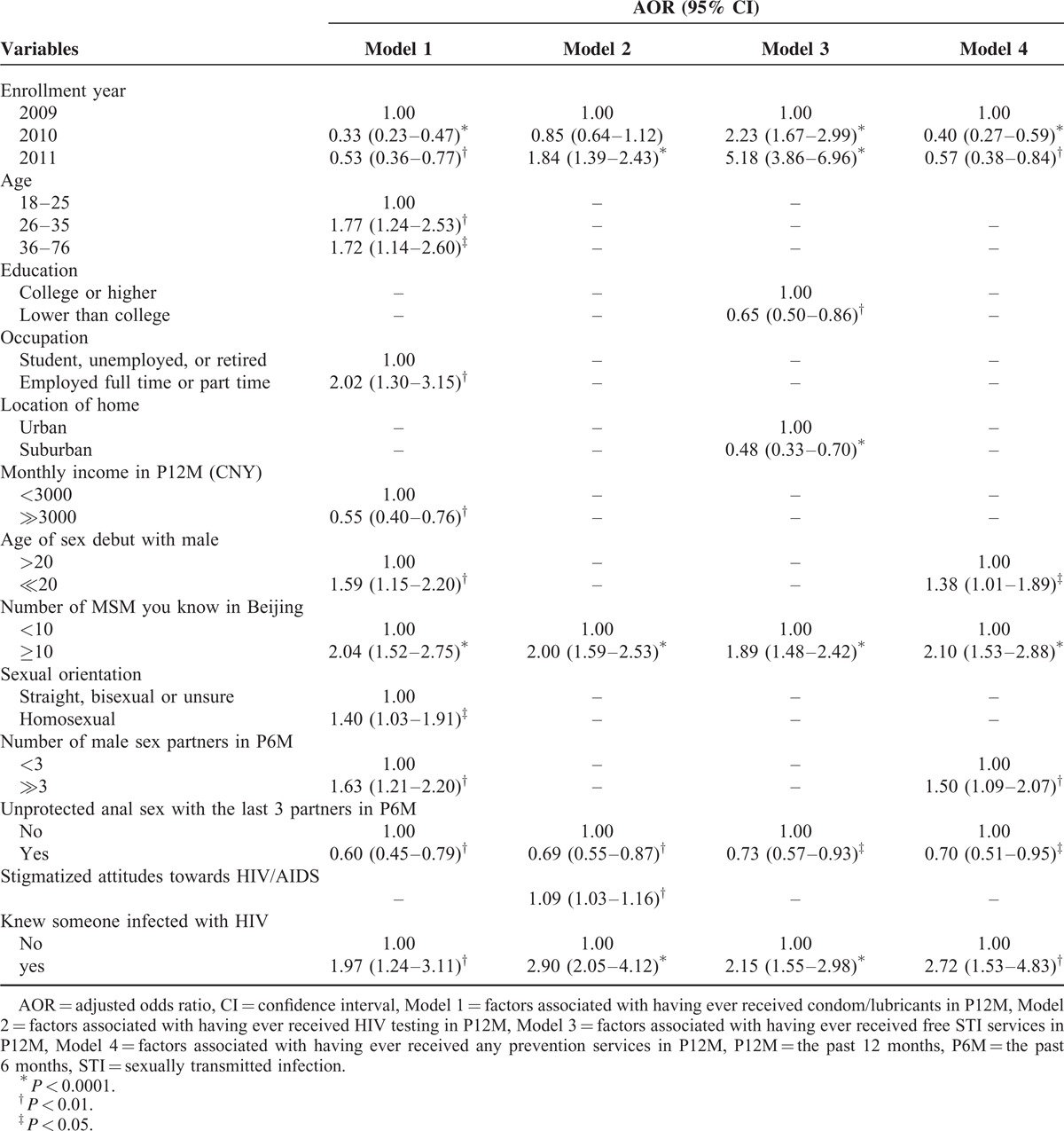
Multivariate Analysis of Association Between Select Variables and HIV/STI Testing and Intervention Services

Participants who reported UAI in P6M (AOR = 0.69, 95% CI: 0.55–0.87, vs no UAI) were less likely to have received HIV testing in the past year in P12M. Receiving an HIV test in P12M was associated with enrollment in 2011 (AOR = 1.84, 95% CI: 1.39–2.43, vs 2009), knowing 10 MSM or more in Beijing (AOR = 2.00, 95% CI: 1.59–2.53, vs<10), with higher stigma scores (AOR = 1.09, 95% CI: 1.03–1.16), and knowing someone infected with HIV (AOR = 2.90, 95% CI: 2.05–4.12). (Table 2, Model2).

Participants who had less than college-level education (AOR = 0.65, 95% CI: 0.50–0.86, vs college or higher education), lived in suburban areas (AOR = 0.48, 95% CI: 0.33–0.70, vs living in urban areas), and had UAI in P6M (AOR = 0.73, 95% CI: 0.57–0.93, vs no UAI) were less likely to have received free STI services in P12M. However, receiving free STI services in P12M was positively associated with being enrolled in 2010 or 2011 (2010: AOR = 2.23, 95% CI: 1.67–2.99; 2011: AOR = 5.18, 95% CI: 3.86–6.96, vs 2009), knowing ≥10 MSM in Beijing (AOR = 1.89, 95% CI: 1.48–2.42, vs <10), and knowing someone infected with HIV (AOR = 2.15, 95% CI: 1.55–2.98) (Table 2, Model3).

### Barriers and Facilitators for HIV Testing and Reasons for not Seeking HIV Testing

The two most commonly cited barriers that prevented participants from having an HIV test were fear of testing positive (79.3%) and perceiving no risk for HIV (75.4%). Almost all participants (99.8%) thought that ensuring confidentiality could encourage more MSM to have a HIV test. Among 407 participants who had previously never been tested for HIV, not knowing where to go for testing (63.2%) and perceiving low risk of HIV infection (55.1%) were the 2 main reasons for not being tested (Table 3).

**TABLE 3:**
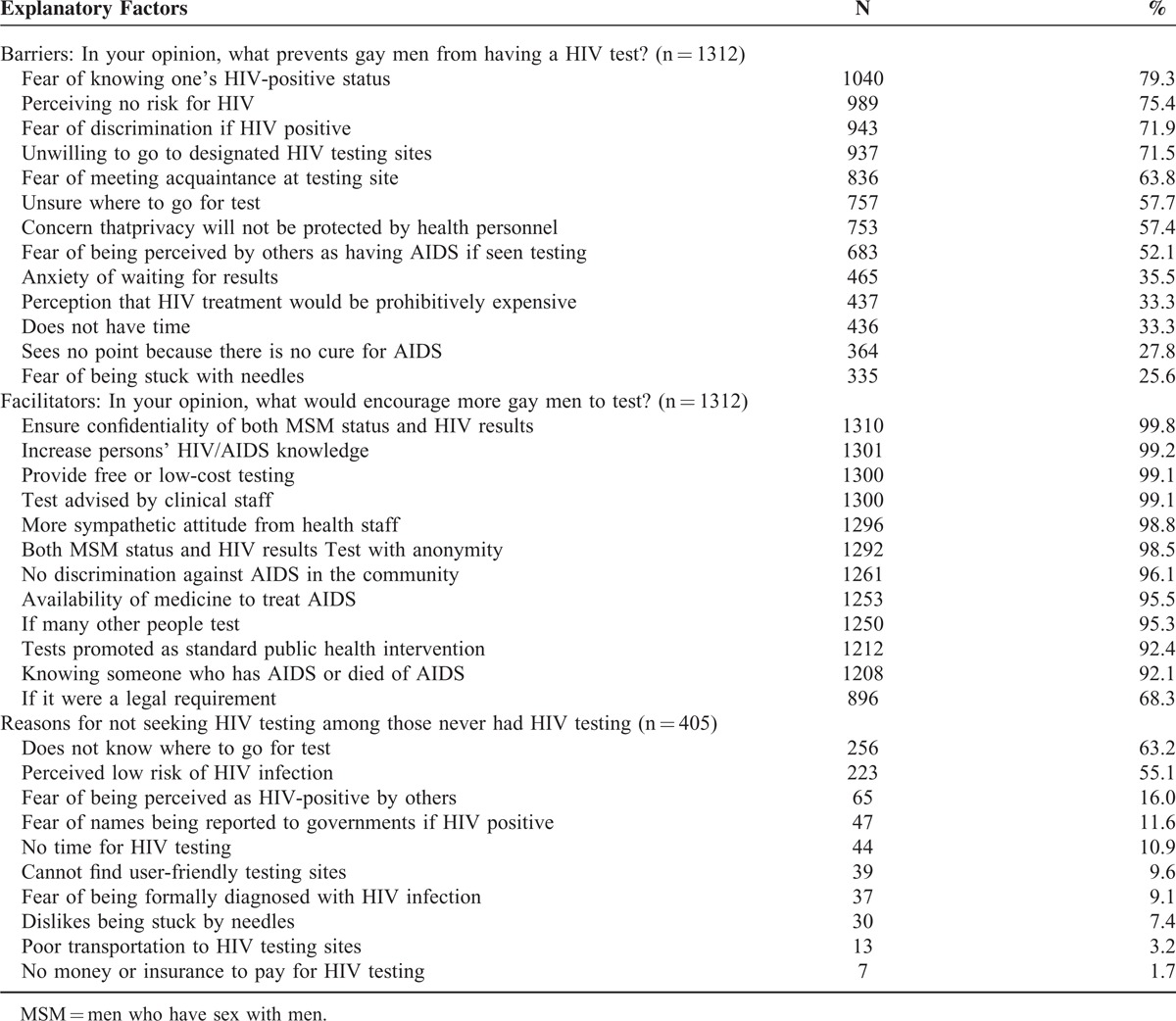
Barriers and Facilitators for HIV Testing and Reasons for Not Seeking an HIV Test

## DISCUSSION

The current study indicated that most MSM in Beijing had received free condom/lubricants (78.7%) in P12M, whereas only about half had received HIV testing (56.2%) or STI services (46.1%) in P12M. Approximately 70% of MSM previously had ever had an HIV test prior to the survey, a prevalence which is comparable with that of a 2009 study of young migrant MSM (aged 18–29 years) in Beijing (72.0%).^21^ Given that HIV and syphilis were more prevalent than in previous studies of Chinese MSM,^3,4,22^ a large number of MSM appear to be at high risk for HIV infection and transmission while remaining unaware of their risks. More efforts are urgently needed to expand the coverage of HIV testing and other prevention services such as STI diagnosis and treatment among this population.

Consistent with findings in previous studies,^23,24^ our research also indicated that MSM with a larger social network (knowing at least 10 MSM in Beijing) were more likely to have accessed HIV testing and other prevention services. In theory, individuals with larger social networks would be more likely to be informed about prevention services. Moreover, men with at least 3 male partners in P6M were more likely to have received free condom/lubricants in P12M, as they might also receive free condom/lubricants that were distributed to their partners. However, those with smaller social networks might have limited opportunities to access these prevention services. Therefore, multiple modes of HIV prevention outreach via mobile apps, Internet, and short message services (SMS) should be promoted to target MSM with few MSM social connections.^25^

Low perceived risk of HIV infection has been hypothesized to be a factor that inhibits people from even considering HIV testing.^26,27^ Similarly, our findings show that 75.4% of participants thought that low perception of HIV risk prevented MSM from having an HIV test, and that more than a half of MSM who had never been tested for HIV cited low risk of infection as the reason for not testing. Although the probability of HIV transmission for anal sex is approximately 18 times greater than that of vaginal sex^28,29^ and 44% of participants were engaged in UAI with at least 1 of their 3 most recent male sex partners in P6M, most participants (71%) still perceived low or no risk of HIV infection. However, it is a bit paradoxical that although most participants perceived low risk of infection, 79.3% of participants reported fear of testing positive as a barrier for HIV testing. Fears of a positive result might be attributed to perceptions of HIV infection as a fatal rather than treatable chronic condition.^30^ Fear of a positive HIV result has been shown to be closely associated with HIV/AIDS-related stigma and discrimination^31^ and was the third most commonly reported barrier to HIV testing in our study. Findings from the current study similarly showed an inverse association between HIV/AIDS stigma and HIV testing in P12M. Therefore, it is not surprising that nearly all participants in our study felt that ensuring confidentiality would facilitate more testing among MSM.

Our study also indicated that MSM who knew someone infected with HIV in the past year were more likely to have received an HIV test as well as other preventive services. One possible explanation may be that knowing HIV-positive MSM may increase one's sense of infection risk within the MSM community, and also reduce HIV/AIDS-related stigma and discrimination.^27^ However, it is also possible that men involved in HIV testing and other preventive services would have more opportunities to know someone infected with HIV who is also engaged in these programs.

UAI in P6M was associated with lower participation for all 3 HIV prevention services. This implies that participation in prevention services such as HIV risk-reduction counseling could help reduce risky behaviors.^7,8^ However, it is also possible that men more conscientious about their personal health were both less likely to engage in risky behaviors and more proactive about taking up prevention services. MSM preventive services have the potential to help MSM reduce risky behaviors and increase health-related awareness.

In China, HIV testing is mainly available through 2 types of government-sponsored programs: HIV VCT clinics and public hospitals. HIV testing is free at the 9000-plus VCT sites mainly established at provincial-, city-, or country-level health departments, but HIV testing supplied in hospitals is generally provider-initiated and not free. Despite the large number of possible testing sites, 63.2% of untested MSM reported that not knowing where to go for a test was one of the reasons for not seeking HIV testing. This finding further underscores the gap between accessibility of testing services and this hard-to-reach population. Future testing strategies should place greater emphasis on universal access to HIV testing and other convenient testing methods such as oral fluid HIV rapid tests.^32^ Moreover, other testing strategies, such as couples HIV voluntary counseling and testing,^33^ rapid home self-testing,^34^ and home dried blood spot specimen self-collection for laboratory testing,^35^ should be considered as promising alternatives to conventional modes of testing.

There are several limitations to this study. First, our findings might not be generalizable to MSM in other cities in China due to sociocultural and regional discrepancies. Second, the cross-sectional nature of the survey prevented us from establishing causal inference. Third, the dual incentive system–financial reward for being interviewed (a primary reward) plus another financial reward for referring others into the study (a secondary reward) may have led to an oversampling of individuals with lower income who may have been more responsive to financial incentives. Finally, though computer-assisted questionnaires likely reduced socially desirable responses, this bias may not have been completely eliminated. Despite these limitations, this study is arguably the first large survey to investigate factors related to HIV testing and prevention services accessibility among MSM in China, and offers guidance for future HIV prevention strategies among MSM in China.

## CONCLUSION

Our findings indicate a high prevalence of HIV, syphilis, and risky behaviors and a relatively low HIV testing rate among MSM in Beijing, implying that current prevention services may be insufficient for containing the ever-expanding epidemics among the MSM population. More efforts are urgently needed to address barriers to HIV testing and improve accessibility of prevention services to those in need.
